# Bèri-Bèri

**Published:** 1895-03

**Authors:** 


*Bèri-Bèri.* By ARTHUR J. M. BENTLEY, M.D. Pp. xii., 245. Edinburgh: YOUNG J. PENTLAND. 1893.

This book consists of a thesis on Bèri-Bèri written for the M.D. degree of Edinburgh in 1889, and a short abstract of some of the more important pathological work done since then, chiefly by Scheube and Baelz and Miura, together with an appendix containing clinical reports of sixty-eight typical cases, in nineteen of which *post-mortem* examinations were made. First of all we will give unqualified praise to the excellent series of photographs which illustrate in the most graphic and satisfactory way the position of the limbs, mode of progression and station in paralysis due to multiple neuritis, the cause of the nervous symptoms of Bèri-Bèri. Then to anyone practising in the tropics the clinical histories in the appendix will be both instructive and practically useful; as a rule the essential features of the different clinical forms of Bèri-Bèri are well brought out; the progress of the cases after admission should, however, have been given in all. We notice in the account of the examination of the urine repeatedly under the heading Phosphates "O" or "a trace": we suppose that this means phosphates precipitated on heating the urine; this should have been clearly stated.

There is a wealth of material in the book, which, however, it requires a good deal of patience and unnecessary trouble to get at. With no wish to find fault, we cannot say that the book is well arranged. It is difficult from it to get a clear idea of the etiology, symptoms, and pathology of the disease. The thesis itself should have been re-written and brought up to date. An attempt is made in an addendum to give some of the recent work on the pathology of Bèri-Bèri, but it is not by any means complete, the author stating that his only acquaintance with the very important work of Pekelharing and Winkler lies in a review in the *Lancet*. Then from the faulty arrangement of the book we read first the author's views as to etiology and pathology, and in addenda other observations completely contradictory to them. These later observations seem, so far as we can judge, to be correct, and to be supported by better evidence and by more thorough methods of work than the author's. For instance, the theory he brings forward that the nervous symptoms are due to changes in the cord seems to be altogether disproved and their dependence on lesions of the peripheral nerves fully established; whilst the influence of diet from the quoted observations of Dr. Takaki, late Director-General of the Tokyo Naval Hospital, would seem to be of very much greater importance in etiology than the author allows.

It seems to us that the author relies on the reader to take the trouble which it was his own duty to take, and that he has not taken sufficient pains to assimilate, digest, weigh, and duly arrange in a clear and readable form the clinical and pathological facts so far as they are known at present, and therefore the work fails in these essential features of a monograph, and in the not less essential ones of completeness and authoritativeness.

In spite of the defects we have indicated, the book contains one of the most complete accounts of Bèri-Bèri to be found in the English language, described by an author who has had a very large experience of the disease.

